# Primary intraperitoneal hydatid cyst presenting with abdominal distension: A case report

**DOI:** 10.1016/j.ijscr.2024.110789

**Published:** 2024-12-26

**Authors:** Bishnu Prasad Kandel, Prajjwol Luitel

**Affiliations:** a Department of Surgical Gastroenterology, Tribhuvan University Teaching Hospital, Institute of Medicine, Tribhuvan University, Kathmandu, Nepal; b Maharajgunj Medical Campus, Tribhuvan University Teaching Hospital, Nepal

**Keywords:** Extrahepatic hydatid disease, Hydatid cyst, Primary peritoneal hydatidosis

## Abstract

**Introduction:**

Peritoneal hydatid disease accounts for 2–13 % of abdominal hydatidosis cases. Due to its nonspecific clinical presentation and potential for multi-organ involvement, the condition is often misdiagnosed. Evidence on managing primary peritoneal hydatid cysts remains limited, presenting challenges in diagnosis and treatment.

**Presentation of case:**

We report a case of primary peritoneal hydatid in a patient, who presented with abdominal distension for a year. Surgical excision and postoperative treatment with albendazole were effective in controlling the disease and preventing a recurrence.

**Discussion:**

The primary goals of treatment are to eliminate the disease, prevent complications, and reduce recurrence risks. Controlled open surgery is preferred for peritoneal echinococcosis to minimize spillage and dissemination. Adjuvant anthelmintic therapy, such as albendazole, prevents recurrence.

**Conclusion:**

In patients presenting with abdominal cystic masses, especially in endemic regions, hydatid disease should be considered in the differential diagnosis. Complete excision of the hydatid cyst combined with antiparasitic therapy is crucial to prevent recurrence.

## Highlights

• Peritoneal hydatid disease comprises 2–13 % of abdominal hydatidosis.
• High index of suspicion is needed for diagnosis.
• Limited evidence exists on managing primary peritoneal hydatid cysts.

## 1 Introduction

Hydatid cyst is a zoonosis caused mainly by the larval stage of the cestode worm *Echinococcus granulosus* [1]. It is endemic in Asia, South America, and certain parts of Africa and in some Mediterranean countries [1]. Humans serve as accidental hosts in the life cycle of *Echinococcus granulosus*. Upon infestation, approximately 95 % of the larvae become lodged in the liver or lungs, while only about 5 % manage to enter the systemic circulation, potentially affecting other organs [2].

The incidence of peritoneal hydatid disease ranges from 2 to 13 % of all abdominal hydatidosis cases [[3], [4], [5]]. There is a high risk of misdiagnosis because of nonspecific presentation with often multiple organ invasion [6]. There is limited evidence regarding the management of primary peritoneal hydatid cysts. Adhering to Surgical CAse REport (SCARE) 2023 guidelines, we report a case of multiple peritoneal hydatid cysts in a 29-year-old patient from an endemic country [7].

## 2 Case presentation

A 29-year-old Nepalese male, with no comorbidities, presented with progressive abdominal distension associated with dull aching non-radiating abdominal pain for one year. He had no history of fever, cough, jaundice, trauma, nausea, vomiting, altered bowel or bladder habits, or significant weight loss. There was no history of close association with dogs. He had not undergone prior surgery. The examination was normal except for a distended abdomen and dullness on percussion leading to a clinical diagnosis of ascites. Hematological tests revealed the following results: hemoglobin: 13.6 g/dL, white blood cell count: 11,500 cells/μL, eosinophils: 4 % (eosinophilia not present), and C-reactive protein (CRP) within normal limits. ELISA for *Echinococcus granulosus* was positive. Liver function and renal function tests were within normal limits.

**Fig. 1:**
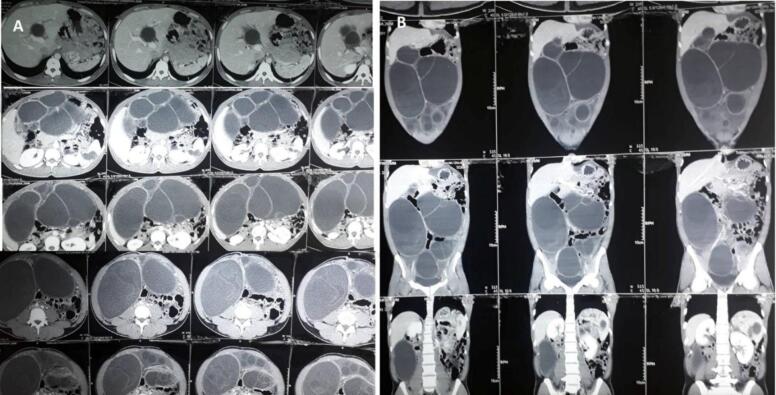
Computed tomography scan of abdomen and pelvis (A: axial, B: coronal section) revealing multiple cystic masses extending throughout the abdomen.

Ultrasonography (USG) of the abdomen revealed multiple, well-defined hypoechoic masses with echogenic septations in the abdomen. A computed tomography (CT) scan of the abdomen and pelvis revealed well-defined hydatid cysts of variable sizes extending throughout the abdomen, with floating membrane in some cysts (Fig. 1).

Chest X-ray was normal. There was no evidence of similar cystic lesions in the liver, lungs, or other organs. Based on the clinical, serological, and radiological evidence, primary multiple intraperitoneal hydatid cysts were diagnosed preoperatively.

**Fig. 2:**
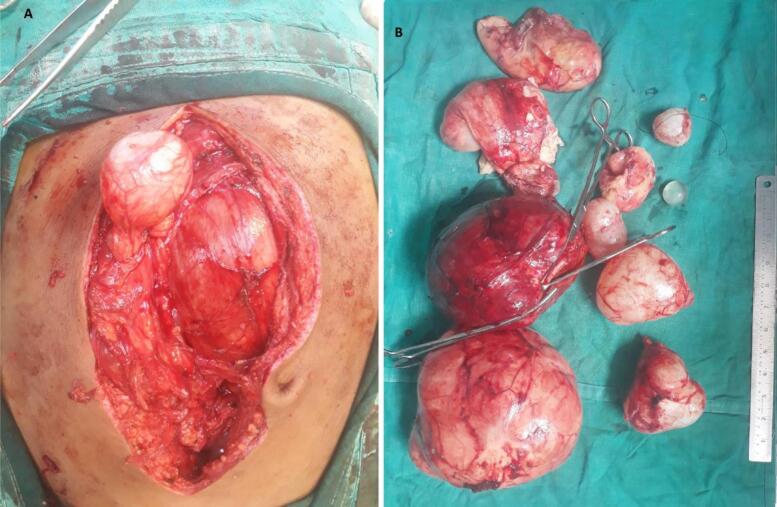
A: intraoperative image showing peritoneal hydatid cyst bulging out through the incision, B: postoperative specimen revealing multiple cysts.

The patient was started on albendazole 400 mg twice daily for one month. After a month, exploratory laparotomy was performed. Intraoperatively, multiple hydatid cysts of various sizes ranging from 2 to 15 cm involving the whole of the peritoneal cavity, and adherent to the peritoneum were present. All the visible hydatid cysts were excised carefully without causing a rupture (Fig. 2).

There were no similar cystic masses in any other abdominal viscera. The patient had an uneventful recovery and was discharged on postoperative day 5. The patient was started albendazole on the third postoperative day and continued for five months. Histopathological analysis confirmed a hydatid cyst. The patient was asymptomatic with no recurrence on chest X-ray and USG after two years of follow-up.

## 3 Discussion

The incidence of peritoneal hydatid disease ranges from 2 to 13 % of all abdominal hydatidosis cases [[3], [4], [5]]. The liver and lungs act as physical barriers, limiting cyst dissemination, which explains the low prevalence of peritoneal echinococcosis [8]. A peritoneal hydatid cyst is classified as primary when no other cysts are detected [3]. The primary type is relatively rare, occurring in only 2 % of cases [9]. However, the exact mechanism of primary peritoneal infection remains unclear, with differing hypotheses proposed in the literature, none of which are definitive [4]. Some studies suggest that dissemination into the peritoneal cavity may occur through systemic or lymphatic circulation, while others propose migration of hepatic cysts or rupture of the adventitia without compromising the cyst membrane [8,9].

Some suggest they occur secondary to spontaneous rupture of a hepatic hydatid cyst or due to the spillage of cystic fluid during intraperitoneal surgery of a primary hepatic, splenic, or mesenteric cyst [3,4]. Differential diagnosis of the intra-abdominal cystic lesions includes mesenteric cyst, gastrointestinal duplication cysts, ovarian cysts, cystadenoma, lymphangioma, abdominal tuberculosis [10]. Specifically in a country like Nepal where abdominal tuberculosis is very commonly seen, they should be considered as differentials for chronic abdominal complaints [10].

Serological testing and imaging play key roles in diagnosing hydatid disease [4]. Among serological tests, ELISA is particularly effective for confirming hydatid cysts, offering a sensitivity range of 95 % to 97 % [11]. Eosinophilia is not a sensitive finding as previous studies reported it in 10.2 % of cases and was similarly absent in our case [12]. Abdominal ultrasonography is typically the first imaging technique to identify the organ of origin and assess the cyst's characteristics with a sensitivity between 90 % and 95 % [11,13]. Computed tomography (CT) is another valuable tool, especially for diagnostic confirmation and treatment planning, boasting a sensitivity of 95 % to 100 % [12]. In our cases, with a clinical diagnosis of ascites, USG was performed, revealing multiple cystic lesions suggestive of hydatid cysts further supported by a CT scan. The combination of imaging findings and clinical assessment, supported by ELISA, helped establish the final diagnosis.

The primary objectives of treatment are to eradicate localized disease, prevent complications, and reduce the risk of recurrence. As performed in our case, controlled open surgery with complete excision of the hydatid cyst is the preferred approach for peritoneal echinococcosis as there is a very high possibility of uncontrolled spillage and peritoneal dissemination of the disease leading to recurrence [12,14]. The guidelines for anthelmintic therapy remain unclear. Reviews have suggested surgical excision with or without adjuvant medical antiparasitic disease is a treatment of choice [12]. As per the CDC recommendations, our case received perioperative albendazole for six months (one month preoperatively and five months postoperatively), and no recurrence was observed.

## 4 Conclusion

A primary hydatid cyst in the peritoneal cavity can develop independently, without involving other organs. When evaluating a patient with an abdominal cystic mass, particularly from endemic regions, hydatid disease should be considered as part of the differential diagnosis. It is also essential to rule out abdominal tuberculosis before proceeding with surgical intervention. To prevent recurrence, the suspected hydatid cyst should be excised with antiparasitic therapy.

## CRediT authorship contribution statement

B.P.K. and P.L. formulated the original manuscript. B.P.K. and P.L. reviewed the final version of the manuscript.

## Consent

Written informed consent was obtained from the patient for publication and any accompanying images. A copy of the written consent is available for review by the Editor-in-Chief of this journal on request.

## Ethical approval

Since this is a case report, our Institutional Review Board of Institute of Medicine (IOM) has waived the requirement for ethical approval for this kind of studies.

Our IRB since processes huge number of articles does not require us to submit application for case report. However, it can demand for the informed consent form which the corresponding author can provide on reasonable request.

## Declaration

All the authors declare that the information provided here is accurate to the best of our knowledge.

## Guarantor

Prajjwol Luitel.

## Research registration number

None.

## Presentation

None.

## Funding

No funding received.

## Declaration of competing interest

All the authors declare that they have no conflict of interest.
